# Faith Leaders Improve Healthy Timing and Spacing of Pregnancy: Results of Operations Research on the Channels of Hope Methodology in Kenya and Ghana

**DOI:** 10.5334/aogh.3944

**Published:** 2023-03-16

**Authors:** Susan A. Otchere, Stephen Omunyidde, Alfonso Rosales, Jacob Ajwang Ochieng, Lilian Chebon, Salome Wumpini Agordoh, Adrienne Allison

**Affiliations:** 1 World Vision, 300 “I” Street NE, Washington, DC, US; 2 Formerly of World Vision, 300 “I” Street NE, Washington, DC 20002, US; 3 Epidemiology at Universidad Evangelica, El Salvador; 4 Columnist, El Mundo newspaper, El Salvador; 5 Formerly of World Vision Kenya, Karen Road, Off Ngong Rd. M/S, Nairobi, 254 Kenya; 6 World Vision Kenya, Karen Road, Off Ngong Rd. M/S, Nairobi, 254 Kenya; 7 World Vision Ghana, Sekyere East Cluster, Effiduase, Ashanti Region, Ghana, US; 8 Formerly of World Vision US, 300 “I” Street NE, Washington, DC, US

**Keywords:** Family planning, pregnancy, Ghana, Kenya, contraceptives

## Abstract

**Background:**

Family planning averts unintended pregnancies, unsafe abortions, and maternal deaths, while improving child health and socio-economic progress, but an estimated 218 million women and girls in low- and middle-income countries, especially in sub-Saharan Africa, have an unmet need for modern family planning. Faith leaders can impact the demand and uptake of family planning. However, there is limited understanding of the mechanisms for effective family planning advocacy by faith leaders. Channels of Hope (CoH) is World Vision’s process that engages faith leaders and faith communities to address health issues.

**Objectives:**

To determine the impact of CoH on promoting healthy timing and spacing of pregnancies and family planning (HTSP/FP) by mothers of children under two years old in select parts of Kenya and Ghana. To also determine faith leaders’ attitudes, perceptions, and potential roles in influencing HTSP/FP after exposure to CoH.

**Methods:**

A mixed methods operations research comprising quantitative (quasi-experimental design with surveys of 4,372 mothers of children under two years old) and qualitative arms (in-depth interviews of 17 faith leaders and their seven spouses) was implemented.

**Findings:**

Taking both countries together, male sterilization, female condom, and LAM were the only FP methods that did not show increases from baseline to endline. Methods with the highest knowledge increases between intervention areas and control areas were implants, injectables and pills, with 18.4, 12.1 and 11.2 percentage point increases, respectively. The faith leaders in both countries reported that their views on healthy timing and spacing of pregnancies changed due to the Channels of Hope workshops.

**Conclusion:**

The HTSP/FP model has potential for positive health and social transformation that is built on the trust of faith leaders. Ghana and Kenya provide great examples of possible scenarios in order to help prepare implementers to scale the learnings of this operations research across sub-Saharan Africa.

## Introduction

Voluntary family planning (FP) has been an essential part of global health efforts for decades, averting 119 million unintended pregnancies, 21 million unsafe abortions, and 134 thousand maternal deaths from July 2018 to July 2019 [1]. FP also improves child health and advances socio-economic progress [2]. Yet, an estimated 218 million women and girls in low- and middle-income countries (LMICs) have an unmet need for modern FP [3]. In sub-Saharan Africa, over 58 million women of reproductive age (WRA) either use no contraception or use traditional methods, with low levels of effectiveness. It is, therefore, important to scale up FP programs that improve the demand and acceptability of modern FP methods. One such approach is the Healthy Timing and Spacing of Pregnancy (HTSP) which helps couples delay, space or limit their pregnancies, consequently improving outcomes for mothers, infants and families [4]. The focus of Healthy Timing and Spacing of Pregnancy messages are on the evidence-based themes of delaying pregnancy until at least 18 years of age, and adequate spacing of a subsequent pregnancy following childbirth or miscarriage, all of which result in substantially improved outcomes for mothers and children [5].

The influence of faith leaders (e.g., imams and pastors) in sexual and reproductive health is well-documented [6,7,8]. HIV/AIDS programs have successfully engaged faith leaders in prevention and behavior change efforts [9,10]. Faith leaders have also been shown to be catalytic forces that can impact demand and uptake of FP commodities and services [11,12]. Their influence is significant, in part, because of their community engagement. Some Africans have reported trust in faith-based leadership, rather than political leadership [13]. Sub-Saharan Africa, a region with a huge unmet need for FP, has examples of faith leaders acting as FP advocates [14,15]. However, the outcomes are varied, and there is very limited understanding of the mechanisms for effective FP advocacy by faith leaders, as well as the success factors that drive those mechanisms. With a grant, World Vision (see **Box 1**) implemented a three-year mixed methods operations research project in selected rural areas of Kenya and Ghana. The project aimed to determine the impact of a faith-based methodology—Channels of Hope (CoH) [16]—on promoting FP acceptability and use by mothers of children under two years old. CoH is World Vision’s model that engages faith leaders and faith communities to address local health-related issues by shifting social norms. Thus, this project also aimed to determine faith leaders’ attitudes, perceptions, and potential roles in influencing reproductive health behaviors after exposure to CoH.

**Box 1 About the implementing organization, World Vision**

World Vision is a Christian humanitarian organization dedicated to working with children, families, and their communities worldwide to reach their full potential by tackling the causes of poverty and injustice.

## Methods

### Study setting

Ghana and Kenya, both multicultural and multireligious, operate democratic governments in the Western Africa and Eastern Africa sub-regions, respectively. Ghana had an estimated 883,000 births in 2019, with about 46% of its FP demand met with modern contraceptives [17]. In the same year, Kenya’s estimated births stood at 1.5 million, with about 78% of its FP demand met [18]. Ghana, with a 2017 maternal mortality ratio (MMR) of 308 deaths per 100,000 livebirths, and Kenya at 342 per 100,000 livebirths, have MMRs that are not as high as other countries in their sub-regions. Yet, Western and Eastern Africa have the world’s highest estimated annual need for investments in sexual and reproductive health care, at $8.2 billion and $6.2 billion USD, respectively [3]. Study sites were Laisamis (intervention) and Isiolo-Oldonyiro (comparison) in Kenya, and West Gonja (intervention) and Zabzugu (comparison) in Ghana. Intervention and comparison sites in each country were selected randomly; there were no explicit criteria for selection.

### Study, aims, and objectives

The study occurred between May 2015 and April 2018, and aimed to measure changes in contraceptive prevalence rate (CPR) and other reproductive health indicators among mothers of children under two in communities where faith leaders received CoH-HTSP processes, versus a comparable community without CoH-HTSP. The hypothesis was that CoH would lead to changes in faith leaders’ attitudes toward HTSP and FP, through increased knowledge and understanding of HTSP and its relation to family well-being; and, through the influential role of these leaders, there would be increased population knowledge, demand, and use of FP. Two research questions guided the study: (1) What is the impact of using the CoH methodology to deliver HTSP messages on the prevalence of contraceptive use among mothers of children under two in rural areas of Kenya and Ghana? (2) Does using the CoH methodology to deliver those messages influence faith leaders to change their own attitudes toward HTSP? Pre- and post-intervention measurements of CPR were carried out at each site using knowledge, practice, and coverage (KPC) questionnaires adapted from the United States Agency for International Development (USAID)’s Maternal and Child Health Integrated Program (MCHIP) [19].

### Study design

**Figure 1:**
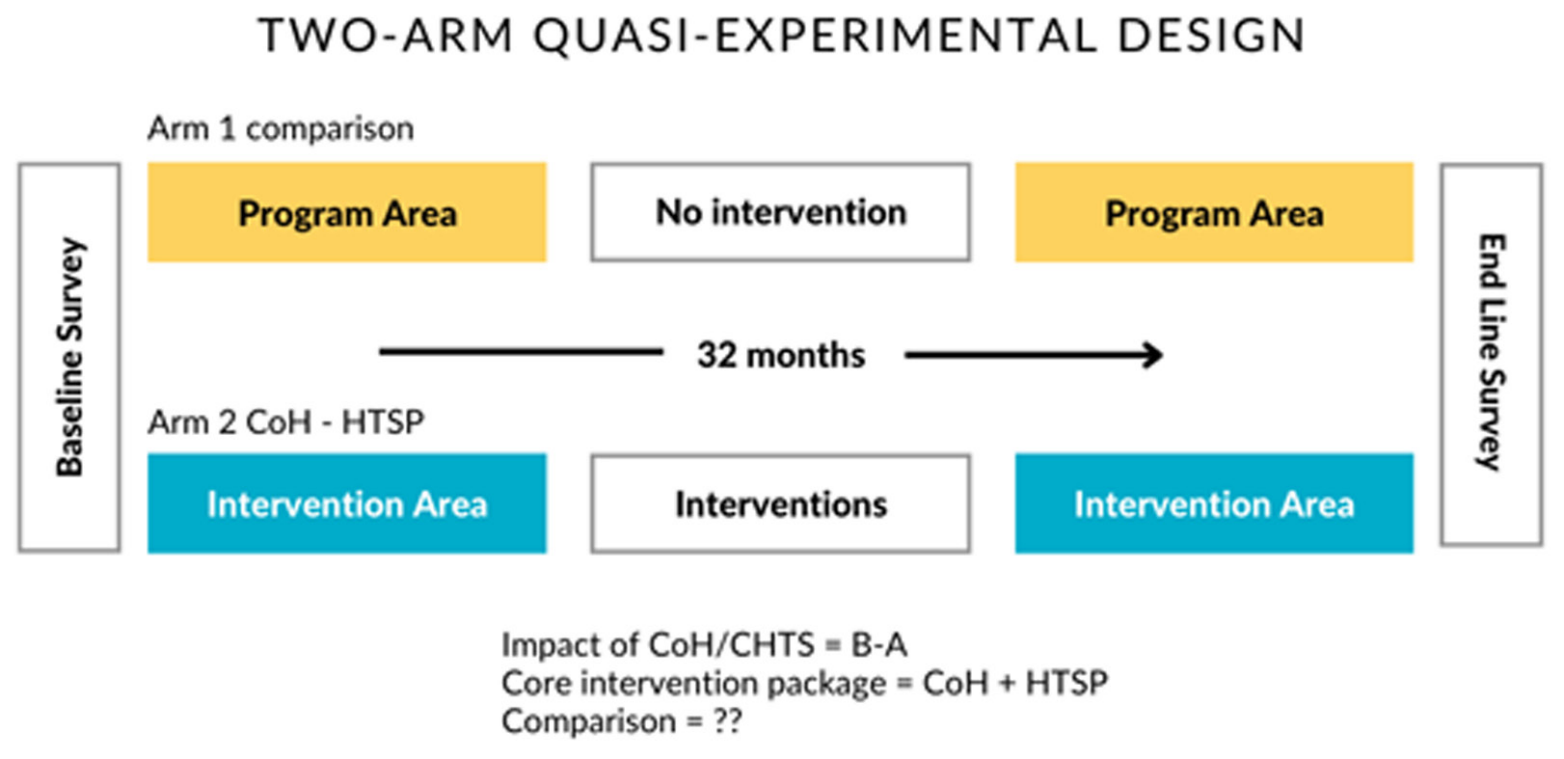
Quasi-experimental design of quantitative method.

We conducted a mixed methods study comprising quantitative (survey questionnaire) and qualitative (in-depth interview) arms. The quantitative method was of a quasi-experimental design (Figure 1), implementing the CoH-HTSP intervention in the experimental group of respondents and comparing results with those without the intervention. Respondents were mothers of children under two residing in the study areas. Several variables were incorporated as part of the study design (Table 1). Following the quantitative research intervention, the qualitative arm was conducted among a sample of CoH participants (faith leaders and their spouses) through in-depth interviews (IDIs). An IDI guide with open-ended questions included the following domains: (1) views on HTSP, fertility, and contraceptive use in the community; (2) experience and views about the CoH workshops; and (3) the faith leader’s role in promoting HTSP in their community.

**Table 1:** Study variables.


INDICATOR	OPERATIONAL DESCRIPTION OF INDICATOR	NUMERATOR	DENOMINATOR

1. Contraceptive prevalence	Percentage of mothers of children ages 0–23 months who are using (or whose partner is using) a modern contraceptive method	Number of mothers of children ages 0–23 months who are using (or whose partner is using) a modern contraceptive method	Number of mothers of children ages 0–23 months

2. Future fertility intentions	Percentage of mothers of children ages 0–23 months who report wanting to wait – at least 2 years from now or after the birth of the child they are expecting – to have another child	Number of mothers of children ages 0–23 months who report wanting to wait – at least 2 years from now or after the birth of the child they are expecting – to have another child	Number of mothers of children ages 0–23 months in the survey

3. Method mix	Percent distribution of modern contraceptive users among mothers of children ages 0–23 months	Number of mothers of children ages 0–23 months who report currently using contraception by a modern method	Number of mothers of children ages 0–23 months who are currently using any contraception

4. Reasons for non-use	Percent distribution of mothers of children ages 0–23 months – who want to either postpone or avoid their next child but are not using a contraceptive method – by reasons for non-use	Number of mothers of children ages 0–23 months who want to either postpone or avoid their next child but are not using a contraceptive method, by reasons for non-use	Number of mothers of children ages 0–23 months who are not pregnant at the time of the survey – and who want to either postpone or avoid their next child – but are not using a contraceptive method

5. Total unmet need for family planning	Percentage of mothers of children ages 0–23 months – who are either (i) pregnant and want to either postpone or avoid their next child, or are (ii) fecund and want to either postpone or avoid their next child – but are not using a contraceptive method	Number of mothers of children ages 0–23 months – who are either (i) pregnant and want to either postpone or avoid their next child, or are (ii) fecund and want to either postpone or avoid their next child – but are not using a contraceptive method	Number of mothers of children ages 0–23 months in the survey

6. Unmet need for spacing	Percentage of mothers of children ages 0–23 months – who are either (i) pregnant and want to postpone their next child, or (ii) fecund and want to postpone their next child – but are not using a contraceptive method	Number of mothers of children ages 0–23 months – who are either (i) pregnant and want to postpone their next child, or (ii) fecund and want to postpone their next child – but are not using a contraceptive method	Number of mothers of children ages 0–23 months in the survey

7. Knowledge of modern family planning methods	Percentage of mothers of children ages 0–23 months who know at least 3 modern methods of family planning	Number of mothers of children ages 0–23 months who know at least 3 modern methods of family planning	Number of mothers of children ages 0–23 months in the survey

8. Knowledge of sources of modern contraceptive methods	Percentage of mothers of children ages 0–23 months who know at least one place or person where they can obtain a modern contraceptive method	Number of mothers of children ages 0–23 months who know at least one place or person where they can obtain a modern contraceptive method	Number of mothers of children ages 0–23 months in the survey

9. Knowledge of adequate birth spacing	Percentage of mothers of children ages 0–23 months who know that a woman should wait at least 24 months after she gives birth before attempting to become pregnant again	Number of mothers of children ages 0–23 months who know that a woman should wait at least 24 months after she gives birth before attempting to become pregnant again	Number of mothers of children ages 0–23 months in the survey

10. Knowledge of benefits of adequate birth spacing	Percentage of mothers of children ages 0–23 months who know one or more benefits of waiting at least 24 months after giving birth before attempting to become pregnant again	Number of mothers of children ages 0–23 months who know one or more benefits of waiting at least 24 months after giving birth before attempting to become pregnant again	Number of mothers of children ages 0–23 months in the survey

11. Knowledge of benefits of delaying a pregnancy until the age of 18 years	Percentage of mothers of children ages 0–23 months who know at least one benefit of a woman delaying a pregnancy until the age of 18 years	Number of mothers of children ages 0–23 months who know at least one benefit of a woman delaying a pregnancy until the age of 18 years	Number of mothers of children ages 0–23 months in the survey

12. Knowledge of increased risk in pregnancies over the age of 34 years	Percentage of mothers of children ages 0–23 months who know at least one health problem that may occur when a woman becomes pregnant when she is older than 34 years.	Number of mothers of children ages 0–23 months who know at least one health problem that may occur when a woman becomes pregnant when she is older than 34 years.	Number of mothers of children ages 0–23 months in the survey

13. Knowledge of increased risk for high parity women	Percentage of mothers of children ages 0–23 months who know at least one health problem that can occur when a woman who has ≥4 children becomes pregnant	Number of mothers of children ages 0–23 months who know at least one health problem that can occur when a woman who has ≥4 children becomes pregnant	Number of mothers of children ages 0–23 months in the survey


### Ethical considerations

Prior to any field work, the study proposal was reviewed and approved by the Navrongo Health Research Center’s Institutional Review Board in Ghana (NHRCIRB234), as well as the Great Lakes University in Kenya Research Ethics Committee (GREC/236/46/2015). A more time-consuming approval process in Ghana led to a shorter (by six months) implementation period there. Informed consent was received from all respondents and interviewees.

### Intervention description

**Figure 2:**
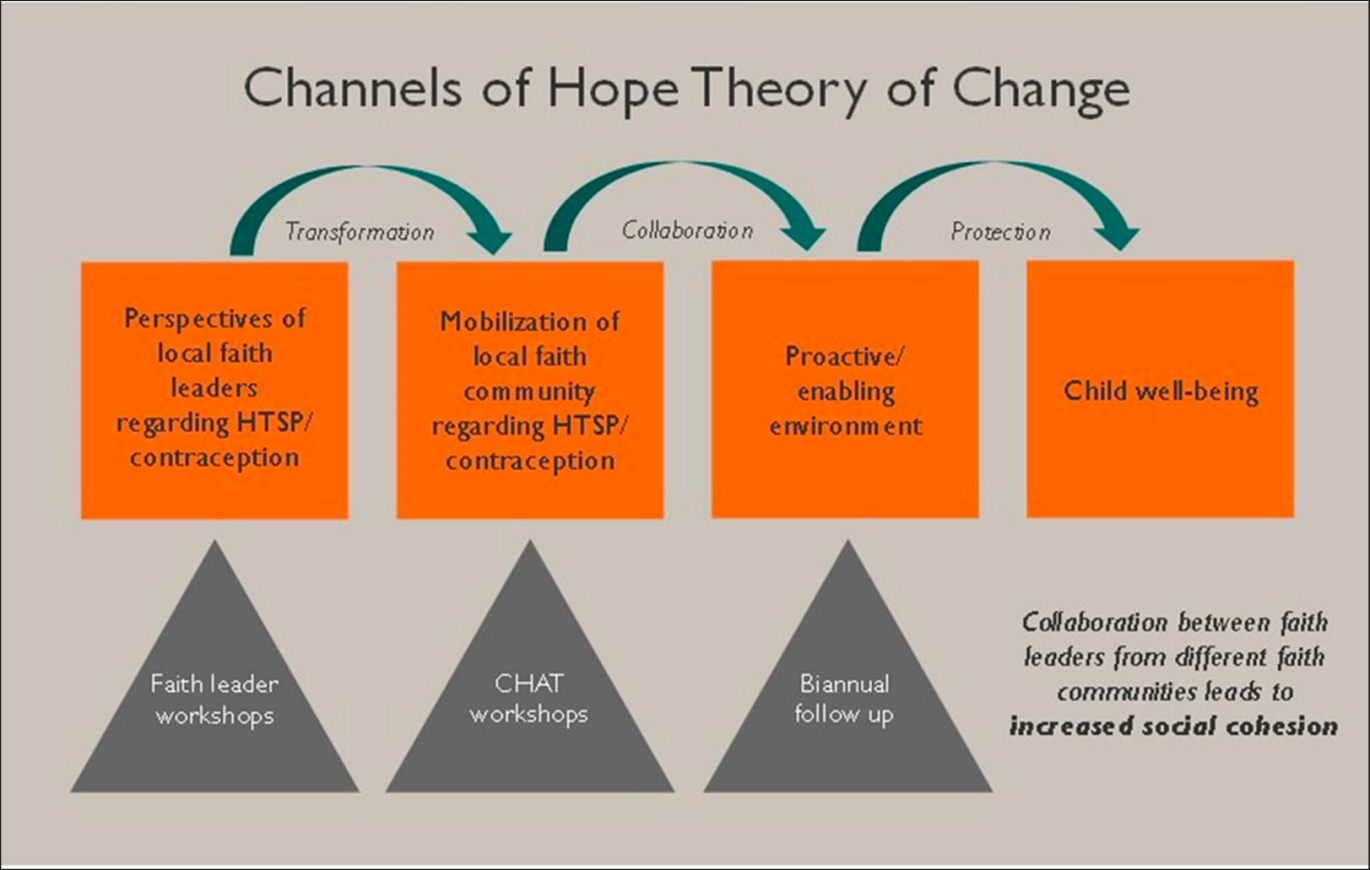
Theory of Change of Channels of Hope model.

Channels of Hope (CoH) is an innovative faith-based model developed by World Vision. It is focused on supporting local faith leaders to become active participants in the health and well-being of their communities through evidence-based information, and insights from their religious texts. The process aims to affect attitudes, norms, values, and practices. There are CoH curricula on several topics, including Gender, HIV/AIDS, and maternal, newborn and child health (MNCH), which includes HTSP/FP [14]. World Vision has used the CoH approach to implement its HTSP programs through faith leaders; however, its effectiveness has not been measured. This operations research is based on a conceptual model that depicts a CoH theory of change (Figure 2) based on social awareness, community mobilization, and creating an enabling environment to produce social change. CoH starts with a series of workshops for faith leaders and other stakeholders structured into the following phases:

1. *Prepare*: This begins with local World Vision staff, Ministry of Health (MoH) stakeholders, and faith leaders who meet to discuss the context, content and methodology of the CoH process, and build capacity of those who will lead the workshops.
2. *Catalyze and strategize*: This equips participants with facts on the topic (in this case, HTSP/FP), scriptural reflection, and an understanding of the community impact and opportunities to engage. Often leading to new attitudes, this phase provides the basis for advocacy and action. The faith leaders then select a small group of other leaders from their congregations to form Congregation or Community Hope Action Teams (CHATs) which work alongside the faith leaders. The members of the CHATs develop their own congregational strategies and action plans, according to their size, vision, and needs.
3. *Empower*: Faith communities implement their action plans in this phase. Action plans outline how the CHATs will deploy HTSP messaging in their communities. One key action item in both Ghana and Kenya was that faith leaders include MNCH/HTSP messages during sermons or faith gatherings. Although CHATs formulate their own plans, the CoH model intentionally guides congregations to engage other relevant stakeholders, including other faith communities and non-faith-based entities.

Throughout the CoH process in this study, World Vision and the MoH collaborated to provide education on HTSP and modern FP methods. Overall, collaboration from government and other partners in convening the workshop served to strengthen understanding of the multi-stakeholder engagement needed to effectively address HTSP. See **Box 2** for HTSP messages used in this project.

**Box 2 Key Messages of Healthy Timing and Spacing of Pregnancies**

**HTSP Key Messages**

1. ***Too young:*** Delay your first pregnancy until at least 18 years old
2. ***Too old:*** Limit pregnancies to a mother’s healthiest years, ages 18–34
3. ***Too close:*** Wait at least 2 years after childbirth before attempting another pregnancy
4. ***Too soon:*** Wait at least 6 months after a miscarriage/abortion before attempting another

## Quantitative Research Arm

### Quantitative data collection

We employed a two-stage random sampling frame to identify households with mothers of children under two. The baseline and final survey adopted the modified KPC household questionnaire, administered on an Open Data Kit (ODK) platform, collecting data from respondents to assess their knowledge, attitudes, and practice of HTSP/FP. Data collection was carried out between December 2015 and January 2018. Combined across intervention and control groups, Kenyan respondents totalled 1,978 and those in Ghana totalled 2,394. At baseline, Kenya had 487 and 499 respondents in the intervention and control groups, respectively; at endline, this was 496 and 496 respectively. At baseline, Ghana had 609 and 579 respondents in the intervention and control groups, respectively; at endline, this was 584 and 622, respectively. Nine mothers from Kenya’s baseline survey were excluded from the analysis due to lack of informed consent.

### Quantitative data analyses

All of the data from the baseline and endline surveys were loaded into STATA 14 software (StataCorp LLC, College Station, TX). Following a careful review of the survey data, variable coding and data transformation were completed. After merging datasets, appropriate numerators and denominators for the identified indicators were computed. Frequencies of the identified indicators were determined and cross-tabulated. The changes from baseline to endline were measured with baseline as reference.

### Results of quantitative data analyses

#### Background characteristics of respondents

Across Kenya and Ghana, a combined total of 4,372 mothers of children under two years were included in the study. Age and gender characteristics of all study respondents are depicted in Table 2. Further, 90% of the mothers were married; 60% had no education; and 80% had less than five years of education. About half of the mothers were housewives, and the other half were involved in either agricultural crops or livestock. About 70% of all mothers combined across countries were Christians. In Kenya, most working women identified as livestock farmers, whereas in Ghana, most identified as crop farmers. In Ghana, almost half of the mothers are Muslim, attending mosques daily; while in Kenya, about 80% identified as Christians, with the majority reporting weekly church attendance.

**Table 2:** Description of all sampled study subjects in Kenya and Ghana combined, by age of mothers, age of children, and sex of children, 2015–2018.


CHARACTERISTICS	BASELINE SURVEY		ENDLINE SURVEY		TOTAL (BASELINE AND ENDLINE)	
		
	NUMBER	PERCENTAGE	NUMBER	PERCENTAGE	NUMBER	PERCENTAGE

Age of Mothers						

<20	167	7.7	139	6.3	306	7.0

20–24	645	29.7	583	26.5	1,228	28.1

25–29	709	32.6	669	30.4	1,378	31.5

30–34	414	19.0	491	22.3	905	20.7

35+	239	11.0	316	14.4	555	12.7

*Total*	*2,174*	*100*	*2,198*	*100*	*4,372*	*100*

Age of the children						

<12 months	1,202	55.3	1,160	52.8	2,362	54.0

12–23 months	972	44.7	1,038	47.2	2,010	46.0

*Total*	*2,174*	*100.0*	*2,198*	*100.0*	*4,372*	*100.0*

Sex of the children						

Girl	1,049	48.3	1,045	47.5	2,094	47.9

Boy	1,125	51.7	1,153	52.5	2,278	52.1

*Total*	*2,174*	*100.0*	*2,198*	*100.0*	*4,372*	*100.0*


#### Knowledge of Contraceptive Methods

We included 11 contraceptive methods in the surveys: female sterilization, male sterilization, intrauterine device (IUD), injectables, implants, oral contraceptives, male condom, female condom, lactational amenorrhoea method (LAM), standard days method (SDM/CycleBeads^®^), and emergency contraceptives. All others (e.g., traditional methods) were grouped under “other method,” yielding a total of 12 method categories under investigation. Respondents reported the highest knowledge on injectables and male condoms, while emergency contraceptives and IUDs were the lowest. Taking both countries together, male sterilization, female condom, and LAM were the only methods that did not show increases from baseline to endline. Kenya experienced higher knowledge increases than Ghana for every method except LAM and SDM/CycleBeads^®^. The increases observed in Kenya ranged from 0.9 percentage points on male sterilization to 24.3 percentage points on implants (Table 3). In Ghana, the highest gains were in LAM, SDM/CycleBeads^®^ and implants. In each country, the proportion of women who had heard of at least three contraceptive methods increased, and this increase was seen in both intervention and control areas. When combining Kenya and Ghana as a whole, methods with the highest knowledge increases between intervention areas and control areas were implants, injectables, and pills, with 18.4, 12.1 and 11.2 percentage point increases, respectively.

**Table 3:** Before and After Comparison in Knowledge on Contraceptive Methods by Country, 2015–2018.


KNOWLEDGE ON	KENYA				GHANA				TOTAL					
		
	BASELINE		ENDLINE		BASELINE		ENDLINE		BASELINE		ENDLINE		TOTAL	
						
	N	% OF N_1_	N	% OF N_2_	N	% OF N_3_	N	% OF N_4_	N	% OF N_5_	N	% OF N_6_	N	% OF N_7_

Female sterilization	209	21.2	414	41.7	721	60.7	770	63.8	930	42.8	1,184	53.9	2,114	48.4

Male sterilization	126	12.8	136	13.7	366	30.8	263	21.8	492	22.6	399	18.2	891	20.4

IUD	214	21.7	275	27.7	474	39.9	500	41.5	688	31.6	775	35.3	1,463	33.5

Injectable	677	68.7	880	88.7	1,056	88.9	1,137	94.3	1,733	79.7	2,017	91.8	3,750	85.8

Implants	429	43.5	673	67.8	822	69.2	996	82.6	1,251	57.5	1,669	75.9	2,920	66.8

Pills	487	49.4	647	65.2	993	83.6	1,097	91.0	1,480	68.1	1,744	79.3	3,224	73.7

Male condom	735	74.5	882	88.9	1,038	87.4	1,062	88.1	1,773	81.6	1,944	88.4	3,717	85.0

Female condom	284	28.8	365	36.8	791	66.6	723	60.0	1,075	49.4	1,088	49.5	2,163	49.5

LAM	611	62.0	457	46.1	304	25.6	434	36.0	915	42.1	891	40.5	1,806	41.3

SDM/CycleBeads^®^	496	50.3	443	44.7	365	30.7	542	44.9	861	39.6	985	44.8	1,846	42.2

Emergency contraceptive	169	17.1	294	29.6	343	28.9	411	34.1	512	23.6	705	32.1	1,217	27.8

Other method	65	6.6	102	10.3	27	2.3	23	1.9	92	4.2	125	5.7	217	5.0

At least three methods	695	70.5	855	86.2	1,068	89.9	1,128	93.5	1,763	81.1	1,983	90.2	3,746	85.7

At least three modern methods	564	57.2	786	79.2	1,061	89.3	1,119	92.8	1,625	74.7	1,905	86.7	3,530	80.7


**%** = Percentage; **N_1_** = Number of women respondents in Kenya, at baseline = **986**; **N_2_** = Number of women respondents in Kenya, at endline = **992**; **N_3_** = Number of women respondents in Ghana, at baseline = **1,188**; **N_4_** = Number of women respondents in Ghana, at endline = **1,206**; **N_5_** = Combined number of women respondents in Kenya and Ghana, at baseline = **2,174**; **N_6_** = Combined number of women respondents in Kenya and Ghana, at endline = **2,198**; **N_7_**= Total number of women respondents in Kenya and Ghana, at baseline and endline, combined = **4,372**.

#### Contraceptive availability and use

Looking at Kenya and Ghana combined, implants were the single group of contraceptives that had the highest increase at endline (36.8%), as a proportion of baseline levels (Table 4). For this method, Kenya’s gain was 50.0% in the intervention areas and 18.5% in the comparison areas. In Ghana, however, this was 58.6% and 12.5%, respectively. Combining data from both countries, there was over three times increase in use of non-modern FP methods. Also combining Kenya and Ghana, in Table 5 we describe observations on several indicators, including methods currently used, and unmet need. Overall, the use of modern contraceptives increased marginally, by about 3.3 percentage points in intervention sites and 1.4 percentage points in comparison sites. However, FP method mix, which denotes modern contraceptive availability and accessibility, showed a major improvement. In all project areas, the indicators of *total unmet need* decreased, highlighting that more women broadly reported they could meet their contraceptive needs. However, *total unmet need for spacing* increased in both intervention and comparison groups, highlighting those women still felt they needed more resources for child spacing, as their children were less than 2 years apart.

**Table 4:** Number and proportion changes of type of contraceptive used in Kenya and Ghana study areas, 2015–2018.


CONTRACEPTIVE METHODS USED	KENYA						GHANA								

	ISIOLO/OLDONYIRO (COMPARISON)			LAISAMIS (INTERVENTION)			WEST GONJA (INTERVENTION)			ZABZUGU (COMPARISON)			TOTAL^†^		
				
	BASELINE	ENDLINE	CHANGE*	BASELINE	ENDLINE	CHANGE*	BASELINE	ENDLINE	CHANGE*	BASELINE	ENDLINE	CHANGE*	BASELINE	ENDLINE	CHANGE*

Injectable	105	115	+9.5%	7	24	+242.9%	62	51	–17.7%	39	58	+48.7%	213	248	+16.4%

Implants	27	32	+18.5%	4	6	+50.0%	29	46	+58.6%	8	9	+12.5%	68	93	+36.8%

Pills	16	11	–31.3%	3	4	+33.3%	26	18	–30.8%	5	11	+120.0%	50	44	–12%

LAM	44	31	–29.5%	185	19	–89.7%	11	7	–36.4%	1	2	+100.0%	241	59	–75.5%

SDM	24	4	–83.3%	16	3	–81.3%	9	8	–11.1%	0	5	N/A	49	20	–59.2%

Male condom	11	1	–90.9%	10	23	+130.0%	5	1	–80.0%	3	1	–66.7%	29	26	–10.3%

Other modern methods	3	2	–33.3%	1	1	zero	3	1	–66.7%	0	0	N/A	7	3	–57.1%

Other method	2	1	–50.0%	3	27	+800%	2	7	+250%	3	6	+100%	10	41	+310.0%

Any FP method	234	196	–16.2%	233	107	–54.1%	140	139	–0.7%	57	92	+61.4%	664	534	–19.6%

Modern CP Mix	162	160	–1.2%	25	58	+132.0%	119	117	–1.7%	53	79	+49.1%	359	414	+15.3%


* As a proportion of Baseline; **N/A** Not applicable, as a proportion of zero cannot be determined; **^†^**Comparison and intervention districts in both Kenya and Ghana, combined.

**Table 5:** Family Planning Methods Used and Needs and Ideal Pregnancy Interval, Benefits and Health Problems-Related Knowledge by Survey and Intervention Status in Kenya and Ghana, combined.


INDICATOR	BASELINE SURVEY						ENDLINE SURVEY					
	
	COMPARISON		INTERVENTION		TOTAL		COMPARISON		INTERVENTION		TOTAL	
					
	N	% OF N_1_	N	% OF N_2_	N	% OF N_3_	N	% OF N_4_	N	% OF N_5_	N	% OF N_6_

Used modern FP method	215	20.0	144	13.1	359	16.5	239	21.4	177	16.4	416	18.9

Future fertility intention	342	31.8	336	30.6	678	31.2	640	57.3	733	67.8	1373	62.5

FP method mix	214	19.9	142	12.9	356	16.4	239	83.0	177	71.4	416	18.9

Total unmet need	325	30.2	260	23.7	585	26.9	532	21.4	610	16.4	1142	52.0

Unmet need for spacing	215	20.0	180	16.4	395	18.2	431	38.6	514	47.5	945	43.0

Knew ≥3 modern FP methods	884	82.1	741	67.5	1625	74.7	971	86.9	934	86.4	1905	86.7

Knew ≥1 source for modern FP (Health Facility/Pharmacist)	729	67.7	678	61.8	1407	64.7	759	67.9	630	58.3	1389	63.2

Next pregnancy ideal waiting time (≥2 years)	493	45.8	490	44.7	983	45.2	848	75.9	897	83.0	1745	79.4

Reported one or more benefits of ≥24 months spacing for next pregnancy	477	44.3	557	50.8	1034	47.6	640	57.3	733	67.8	1373	62.5

Mentioned at least one benefit of age ≥18 years for first pregnancy	602	55.9	731	66.6	1333	61.3	710	63.6	777	71.9	1487	67.7

Mentioned at least one health problem for pregnancy after age 34 years	568	52.7	666	60.7	1234	56.8	764	68.4	826	76.4	1590	72.3

Mentioned at least one health problem for pregnancy after having four children	378	35.1	280	25.5	658	30.3	227	20.3	225	20.8	452	20.6


**n** = Number; **%** = Percentage; **N_1_** = Number of women respondents at baseline in comparison districts in Kenya and Ghana combined = **1,077**; **N_2_** = Number of women respondents at baseline in intervention districts in Kenya and Ghana combined = **1,097**; **N_3_** = Number of women respondents at baseline in comparison and intervention districts in Kenya and Ghana combined = **2,174**; **N_4_** = Number of women respondents at endline in comparison districts in Kenya and Ghana combined = **1,117**; **N_5_** = Number of women respondents at endline in intervention districts in Kenya and Ghana combined = **1,081**; **N_6_** = Number of women respondents at endline in comparison and intervention districts in Kenya and Ghana combined = **2,198**.

#### Knowledge of HTSP key messages

The proportion of total respondents correctly citing healthy pregnancy spacing of at least two years increased by 38.3 percentage points in the intervention areas and by 30.1 percentage points in comparison areas. The proportion of respondents mentioning at least one benefit of delaying a first pregnancy until at least 18 years old increased by only 5.3 percentage points in the intervention groups, and 7.7 percentage points in the comparison group.

## Qualitative Research Arm

### Qualitative data collection

Each of the eight interviewers (four from each country) were trained in qualitative in-depth interview (IDI) techniques. Study participants were contacted by the project for interview appointments. A total of 24 participants who completed the CoH workshops were purposively selected. The sample comprised eight faith leaders and four spouses in Ghana; and in Kenya, nine faith leaders and three spouses. The interviews were conducted in the native language over a period of three weeks. The interviews were then transcribed, translated, and reviewed for quality assurance.

### Qualitative data analyses

Transcripts were transferred to ATLAS.tiv7 (ATLAS.ti Scientific Software Development GmbH, Germany) for coding and analysis. A grounded theory approach was taken in analyzing the data, by first assigning freely emerging codes to sections of the interviews and later comparing codes across interviews. Several codes were subsumed under a super code which corresponded to the domains covered in the questionnaire. Within each domain, similarities and differences were examined and explained.

### Results of qualitative data analyses

#### Characteristics of Respondents

Participants of the in-depth interviews ranged from 21–63 years of age. In Ghana, all had some form of education, with three completing university. All but two of the participants were married. Their family sizes ranged from 2–11 children, with most (75%) of the participants reporting having ≥ 4 children. In Kenya, four had received higher education, including two who completed Arabic studies. Only two participants were single. The number of children in each of their families ranged from 2–12, with the majority reporting ≥ 4 children.

#### Views on HTSP

The respondents in both countries reported that their views on HTSP changed due to the CoH workshop. Several reported they were most influenced by the economic aspect of having multiple children. Many, therefore, started looking at their large families differently, leading some to consider using contraception. Women, in general, cited the health of the mother and the rising cost of rearing a child as key reasons for delaying pregnancies.

*“HTSP will allow the mother enough time to regain her strength after the delivery. It also allows the mother to breastfeed her baby well so that the baby also becomes very healthy and strong. Again, HTSP allows the family to save money because you would not be visiting the hospital frequently because of ill health—so you are able to save enough money to do other things.”*

#### Family Size

Participants in Ghana spoke of the ill effects of having too many children due to the expense of rearing them. Only a few respondents (three) had a favorable attitude toward large families. Overall, Kenyan participants were concerned about the inability of families with many children to provide good care for them due to economic hardship.

#### Family Planning

Faith leaders spoke positively of FP and credited the CoH workshop for having a positive impact on their beliefs. After the workshop, most started thinking of modern FP methods as complementary to natural contraception, which was endorsed by their respective religions.

*“To be truthful, before the CoH workshop, I knew about family planning, but I never used it because of my belief as a Muslim faith leader. But when we went to the workshop and they educated us on family planning, I accepted it and added it to my daily life to help myself, my family, and those that I lead in the community.”*

All respondents reported a more favorable attitude toward FP methods such as condoms, pills and injections. They cited that many of their earlier misconceptions, such as harmful effects of modern methods, were addressed at the CoH workshop. Two faith leaders reported that the only challenge from the workshop was the idea of teaching safe sex and condoms use to youth. They acknowledged their struggle with the belief that it would encourage sexual activity.

#### Perceived Role of Faith Leaders

Most faith leaders affirmed their influence in their communities and expressed the importance of their role in promoting HTSP.

*“I felt very happy to be a part of the CoH project because it will help me as a faith leader to better educate my people and especially the youth on how to space their children when they get married. It will also help our wives with regards to their health and afford the men the opportunity to take good care of the children. It will guide me personally on how to space my children…”*

A few participants were cautious about the impact of their messages to the community and felt it may be challenging as all elders would not take kindly to their teaching. Overall, participants were happy with the detailed and factual information provided through the workshop.

## Discussion

The FP literature has grown significantly over the past three decades, with important contributions to programming, policy, and practice. Our research is a significant addition to the discourse on faith-based approaches to optimize acceptability and use of modern contraceptive options. We found that improving delivery of HTSP/FP messages in Kenya and Ghana generally increased demand and use of modern contraceptive methods, as CoH-HTSP significantly increased unmet need in intervention areas. Our results show that the engagement of faith leaders in the design and delivery of these messages proved successful, and these findings agree with findings from previous work done in Kenya [20], as well as other countries in sub-Saharan Africa [21,22]. However, our results also showed that increasing knowledge of contraceptive methods and the importance of adequate spacing are insufficient to achieve HTSP. There is also the need for the faith community to directly or indirectly catalyze access to quality family planning services, documenting uptake and contraceptive use, and ensuring the provision of these services occur within resilient and strengthened health delivery and supply chain systems.

Our results also showed some disparate results among different FP methods, and across different areas of the two study countries. Specifically, in both intervention and comparison sites combined, Kenya showed a higher increase in the knowledge of at least three modern methods of contraception than Ghana did (Table 3). However, while the proportion of women with knowledge of other (non-modern) methods increased in Kenya, it marginally reduced in Ghana. This apparent paradox must be considered in light of the results from the proportion of respondents *using any FP method*. Here, we observe (Table 4) that while Kenya showed decreases in both intervention and comparison groups, Ghana showed a 61.4% increase in its comparison group, with almost no change in its intervention group. Factors responsible for this inter-country difference are likely contextual. While Kenya’s population is almost double that of Ghana’s, the former has 78% of its demand for FP met by modern methods, while in Ghana, it is only 46% [17,18]. Other authors have also found counter-directional results in the FP landscape of Kenya and Ghana that could be explicable by each country’s health system. For example, an analysis of publicly available data showed that the odds of contraceptive use were higher among Kenyan women who lived in settings that required user-fee for contraceptive services, but lower among Ghanaian women residing in such areas [23]. Understanding the reasons for this would require appraisal of other economic indicators of each country. We note that, prior to this study, both countries had recognized the facilitatory roles which religious or faith leaders play in improving FP and, broadly, MNCH [24]. Nonetheless, experimental or quasi-experimental approaches to appraise the impact of such roles on contraceptive use are sparse. To this end, our work aimed to address such gaps.

Assessing for important knowledge items in Kenya and Ghana together, we found increases in the proportion of respondents who knew ≥ 3 modern FP methods in both comparison and intervention sites. However, there was barely any change in the proportion of respondents who knew ≥ 1 source of modern FP. Assessing use of different contraceptive options, Kenya showed a much higher increase in injectable use, especially in intervention areas, compared to Ghana. Both countries showed higher implants use in their respective intervention sites, relative to comparison sites. A higher proportion of women in Kenya’s intervention site (more than Ghana’s) reported decreases in the use of the lactational amenorrhea method (LAM), while the comparison site in Ghana reported increases in LAM. Though Ghana-specific assessments of LAM knowledge are sparse in the literature, groups of women in other countries recognized the contraceptive benefits of breastfeeding, but specific knowledge of LAM was minimal and varied [25,26]. Future studies measuring associations between knowledge of FP methods and the acceptability of same could further buttress our findings. A systematic review reported shorter breastfeeding as one of only two factors that were consistently associated with a short birth interval [27]. Such findings suggest opportunities for LAM to be better ingrained in advocacy efforts as a complementary FP method approach to modern methods.

On assessing the understanding and retention of the HTSP/FP messaging content, following delivery by the faith leaders in the intervention sites in Kenya and Ghana combined, we found that there was an increase of 38.3 percentage points in the number of respondents in these sites who could state the ideal pregnancy interval (≥ 2 years). This was higher than the 30.1 percentage points change seen in comparison sites. Also, the percentage of the respondents that could state at least one benefit of delaying a first pregnancy until after age 18 increased by 5.3 percentage points in the intervention sites, less than the 7.7 percentage point increase seen in comparison sites. Finally, our study findings seem to indicate that the attitudes among Muslim and Protestant Christian faith leaders are positive toward reproductive health, including approval of modern contraceptive methods. These findings align with research from the Georgetown University Institute for Reproductive Health, which demonstrated strong support among faith leaders for church involvement to expand FP programs [28], with most participants (similar to our study) associating contraception with positive links to improving maternal and child health and poverty reduction. Likewise, a study in Malawi highlights the benefits of a faith-based health promotion platform, such as CoH [14].

### Strengths and limitations

The quasi-experimental design of this study is its main strength. However, the non-random assignment into treatment or comparison groups limits the internal validity and generalizability of its findings. Another limitation of the study is the inability to assess for confounding or mediation, because of a lack of information on pre-existing characteristics of the respondents in the separate intervention and comparison groups. We found that some faith leaders were hesitant to use the pulpit as a delivery platform. It has been documented that participation and support from church leadership is vital to the success of the intervention [29,30]. The implementation design of our intervention did not involve church leaders at the national level. This recognition of the most influential voice within the religious structure might be a design step within the CoH model that should be considered. A limitation of the qualitative arm was the small sample size and the lack of a baseline survey from this population.

### Recommendations

Deploying the HTSP/FP approach across areas of Ghana and Kenya, utilizing the CoH methodology, has been shown to yield improved knowledge, acceptance, and potential use of modern family planning methods. Reproductive health professionals in sub-Saharan Africa might, therefore, consider contextualizing this approach for use in their program design and implementation, social accountability efforts, service delivery activities, and policy advocacy to governments. Examples of immediately actionable recommendations that could enable women achieve adequate birth spacing include governments and reproductive health program managers incorporating CoH methodology into social and behavior change communication programs that complement family planning projects and services, especially in underserved communities; ensuring that faith leaders are visible at all stakeholder engagements for the planning, implementation, and review of all reproductive health programs and policies at national and subnational levels; and broadening the methods mix of family planning commodities remain a top priority for reproductive health policy and program professionals. As HTSP/FP is a veritable strategy for the improvement of the health and socio-economic well-being of families, faith leaders and religious institutions should be integral to these efforts. However, the involvement of faith communities must not simply be an “add-on” to public health strategies, but fundamental to the multi-stakeholder approach necessary for sustained improvement in maternal and child health outcomes. Faith institutions should also be supported to deliver reproductive healthcare, including contraception and preventive healthcare. More research to strengthen the design and effectiveness of the CoH intervention may be useful in contextualizing and scaling up this approach. Testing the CoH model against other community interventions (both faith-based and non-faith-based) aimed at HTSP/FP is an opportunity for stakeholders at all levels to further determine the impact of CoH.

## Conclusion

The current study demonstrated that the application of the CoH model to increase knowledge of HTSP as well as the demand for modern contraceptives in rural areas of Ghana and Kenya is an acceptable and effective methodology. The CoH intervention influenced faith leaders to positively change their own attitudes toward HTSP/FP. This, therefore, highlights the potential of an innovative faith-based approach toward positive health and social transformation. However, increasing knowledge of contraceptive methods and the importance of adequate spacing is insufficient to achieve HTSP. Effective integration of the evidence from Ghana and Kenya can spur a range of possible strategies to mainstream and scale the learnings of this operations research in similar settings. With the COVID-19 pandemic still lingering and disrupting FP supplies and services [31], evidence from Kenya [32] show that most (81.6%) women did not change their contraceptive status due to the pandemic, and for those who changed, they were more likely to change to a new method than to discontinue. As such, there appears to be an opportunity to scale of HTSP/FP programs in Kenya, through faith leaders.
